# “Quality over quantity:” smaller, targeted lesions optimize quality of life outcomes after MR-guided focused ultrasound thalamotomy for essential tremor

**DOI:** 10.3389/fneur.2024.1450699

**Published:** 2024-11-13

**Authors:** Vivek P. Buch, David Purger, Anjali Datta, Allan Wang, Daniel Barbosa, Yosefi Chodakiewitz, Lior Lev-Tov, Chelsea Li, Casey Halpern, Jaimie Henderson, Jennifer A. McNab, Rachelle R. Bitton, Pejman Ghanouni

**Affiliations:** ^1^Department of Neurosurgery, Stanford University, Stanford, CA, United States; ^2^Department of Neurosurgery, Perelman School of Medicine, University of Pennsylvania, Philadelphia, PA, United States; ^3^Department of Neurosurgery, Rambam Health Care Campus, Haifa, Israel

**Keywords:** MRI-guided focused ultrasound surgery, quality of life, thalamotomy, essential tremor, normative tractographic atlas

## Abstract

**Introduction:**

MRI-guided focused ultrasound (MRgFUS) thalamotomy of the nucleus ventralis intermedius (VIM) has emerged as a powerful and safe treatment modality for refractory essential tremor. While the efficacy of this technique has been extensively described, much remains unclear about how to optimize MRgFUS for patient quality of life (QoL), which may depend as much on a patient’s adverse effect profile as on the magnitude of tremor suppression. Diffusion tensor imaging (DTI) has been used to help guide targeting strategies but can pose certain challenges for scalability.

**Methods:**

In this study, we propose the use of a simplified patient-reported change in QoL assessment to create an unbiased representation of a patient’s perception of overall benefit. Further, we propose a large-sample-size, high-resolution, 7 T DTI database from the Human Connectome Project to create a normative tractographic atlas (NTA) with representations of ventral intermediate nucleus subregions most likely to be structurally connected to the motor cortex. The NTA network-based hotspots are then nonlinearly fitted to each patient’s T1-weighted MRI.

**Results and discussion:**

We found that smaller lesion size and higher extent to which the lesion is within the NTA hotspot predicted patients’ change in QoL at last follow-up. Though long-term change in clinical rating scale for tremor (CRST) impacted QoL, neither intraoperative tremor suppression nor the patient’s long-term perception of tremor suppression correlated with QoL. We provide an intraoperative threshold for accumulated dose volume (<0.06 cc), which along with the network-based hotspot in the NTA, may facilitate an easily scalable approach to help limit treatment to small, safe yet effective lesions that optimize change in QoL after MRgFUS.

## Introduction

Essential tremor (ET) is the most common movement disorder, estimated to affect approximately 5–6% of adults over the age of 60 (1). Up to half of patients remain debilitated despite medical management (2, 3, 56), leading to referral for treatment with deep brain stimulation (DBS) or thalamotomy targeting the nucleus ventralis intermedius (VIM) of the thalamus, a sensorimotor integration center connecting the cerebellum to cortical motor pathways (3–5).

MRI-guided focused ultrasound (MRgFUS), an incision-less approach, is increasingly used to treat essential tremor of the hands. While prior studies have established improvement in tremor from and the safety profile of MRgFUS thalamotomy (6–8), less emphasis has been placed on overall post-procedural quality of life (QoL), which likely reflects a subjective combination of tremor relief, freedom from debilitating side effects, and overall impact of the procedure. The relationship between tremor control and QoL can be highly variable between different cohorts and studies (9–15), likely driven by a patient-specific subjective balance between the impacts of tremor suppression and potential side effects on a patient’s quality of life. For example, ataxia, the most frequent side effect after MRgFUS thalamotomy, may be sustained in 18% of patients long term (6); such a side effect may outweigh alleviation of tremor in patients’ own assessment of their overall treatment-induced change in QoL. The Fahn-Tolosa-Marin Clinical Rating Scale for Tremor (CRST) typically used to quantify the results of MRgFUS thalamotomy (57) does not address patient satisfaction. The Quality of Life in Essential Tremor (QUEST) questionnaire (16) asks about QoL only in terms of the impact of tremor on specific daily functions and overall health status. Without a more directed question about treatment-induced change in QoL, patients may not know to report the impact of any non-tremor related effects on current QoL. This introduces a potential limitation for understanding the relative impact of tremor suppression versus non-tremor related effects on quality of life. Therefore, we introduced a simplified, patient-reported impression of change in overall QoL after their procedure to measure how patients qualitatively assess the impact of the procedure as a whole.

Traditionally, MRgFUS thalamotomy targeting has relied on canonical, indirect targeting to estimate the location of the VIM nucleus, as it is not readily visible on MRI (17). Due to uncertainty regarding the location of the VIM, patients are kept awake and frequently examined to solicit immediate clinical feedback, facilitating rapid adjustment of the target (18). With this approach, if ablation at the canonical target produces incomplete tremor suppression, then target adjustment is based entirely on clinical feedback, increasing lesion size and prolonging procedure time. Patient-specific diffusion tensor imaging (DTI) can optimize targeting (4, 19, 20, 58) however, high-resolution DTI is technically challenging to acquire, and non-uniform fiber tracking algorithms across both deterministic and probabilistic approaches may lead to a lack of reliability and overall accuracy (21), which may contribute to difficulty with scaling this approach across academic and non-academic hospital settings. Here, we propose the use of a large-sample-size, high-resolution, 7 T DTI database from the Human Connectome Project to create a normative tractographic atlas (NTA) to identify VIM subregions likely to be structurally connected to the motor cortex. The NTA network-based hotspots are then nonlinearly fitted to each patient’s imaging. In a retrospective cohort, we investigated the relationships between MRgFUS treatment-related QoL change and lesion characteristics, as well as the extent to which the lesion fell within the patient-fit NTA hotspot.

## Methods

### Patient selection

This study included 60 patients who were treated with commercial (post-FDA-approval) MRgFUS ablation for disabling upper extremity tremor at Stanford University prior to July 2020, before implementation of some of the advanced targeting techniques highlighted, enabling unbiased review of clinical QoL outcomes and lesion/hotspot characteristics. Medical records and imaging were retrospectively reviewed and processed. Inclusion criteria included age at least 18 years, diagnosis of ET with or without Parkinsonian features confirmed by a movement-disorders-trained neurologist and the treating neurosurgeon, and post-treatment follow-up of at least 90 days. Patients without a preoperative noncontrast magnetization-prepared rapid gradient-echo (MPRAGE) scan acquired at 3 T were excluded (see Table 1 for imaging parameters). This study was approved by the Stanford University Institutional Review Board.

**Table 1:** MRI sequence parameters.

	Preoperative MPRAGE (BRAVO or T1-FFE)	Postoperative T2-weighted CUBE	Postoperative FGATIR
Echo time (or Effective Echo Time)	3.0–3.5 ms	84–96 ms	3.9–5.4 ms
Repetition time	7.9–8.2 ms	2,502 ms	9.7–12.7 ms
Inversion time	400 ms		300 ms
Echo train length		100	
Flip angle	8-13^o^	90^o^	7^o^
Reconstructed matrix size	512 × 512 × 170–344	512 × 512 × 121	512–568 × 512–568 × 178–232
Field of view	240 × 240 × 170–188 mm	240 × 240 × 242 mm	200–260 × 200–260 × 155–178 mm

The typical parameters used to acquire and reconstruct the preoperative T1-weighted MPRAGE and postoperative T2-weighted CUBE and FGATIR images in the patients in this study.

### Tremor suppression and QoL assessment

Participants were seen for a preoperative visit, where their symptoms and the effects on activities of daily living were evaluated using the Fahn-Tolosa-Marin Clinical Rating Scale for Tremor (CRST; Fahn et al., 1988). On the day of treatment, CRST parts A (limited to tremor amplitude) and B were repeated immediately prior to treatment, with part B being repeated after each ablative sonication and at the end of the treatment session. The part B assessment included drawing an Archimedes spiral, drawing three straight lines, and writing their name. MRgFUS therapy was delivered according to standard-of-care treatment guidelines as outlined in Elias et al. (7, 22). Lesion characteristics, including accumulated dose volume (in cc), were recorded. Participants were reached for an initial telephone call an average of 4.89 ± 1.35 days after their procedure, followed by up to two additional calls in the weeks after the day of treatment to assess for rapid development of side effects. Participants were then seen in the clinic for a first follow-up visit an average of 144 ± 21 days after the day of treatment, followed by up to two additional clinic visits; CRST assessments were repeated at in-person clinic appointments. Participants were discharged from the study after three follow-up visits without a need for ongoing treatment, after they could no longer be reached for further follow-up, or at the end of study enrollment, whichever occurred last. Because the Clinical Rating Scale for Tremor typically used to quantify the results of MRgFUS thalamotomy does not address patient satisfaction, and because the Quality of Life in Essential Tremor (QUEST) questionnaire (16) asks about QoL only in terms of the impact of tremor on overall health status, we introduced a simplified, patient-reported, subjective change in QoL after their procedure to measure how patients qualitatively assess the impact of the procedure. At last follow-up, participants were asked to holistically assess their quality of life compared to before treatment in terms of tremor relief, side effects, and impacts on activities of daily living, and choose “better,” “approximately the same,” or “worse”. Any adverse effects experienced were documented at each postoperative contact.

### Lesion segmentation

Approximately 30 min after treatment (after removal of the ultrasound transducer helmet), MRI, including volumetric T2-weighted fast spin echo (CUBE) and fast gray matter acquisition T1 inversion recovery (FGATIR) (3 T Discovery MR 750, GE Healthcare) sequences (Table 1), were acquired using an 8-channel head coil. These MRIs were manually segmented using ITK-SNAP software (23). Zones I and II, corresponding to durable lesions, were segmented; zone III, corresponding to vasogenic edema (24), was excluded.

### Normative tractographic atlas creation

**Figure 1:**
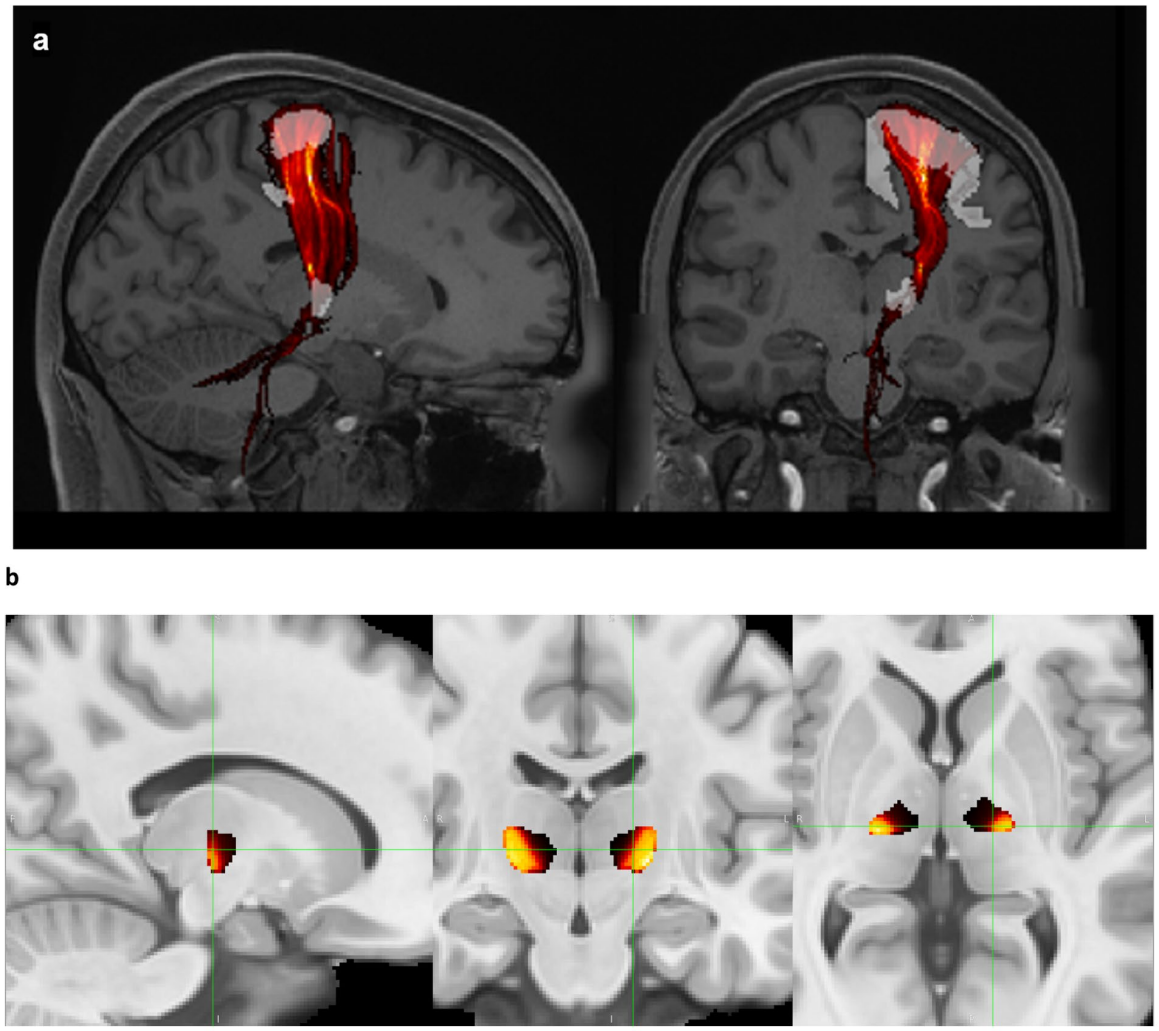
Normative tractographic atlas. (a) VIM-precentral streamlines (red, thresholded and displayed as a maximum intensity projection) connect the brainstem, cerebellum, VIM (white), and motor cortex (white) in a subject in the Human Connectome Project 7 T database. (b) Normative VIM-precentral objects (the seeds most likely to project to motor cortex) in hot colors within the entire VIM (black) in MNI space. In MNI space, the green crosshairs are at the medial apex of the hottest voxels (15 mm lateral to midcommissural plane, 1 mm anterior to the 25% ACPC distance from PC, and 2 mm superior), which represents the MNI-space target for MRgFUS due to the predominant inferoanteriolateral spread of sonication energy. Exact coordinates of this point vary for each patient based on the nonlinear transform back to individual native space.

Normative tracts were identified using probabilistic tractography on high-resolution 7 T diffusion data from the Human Connectome Project (HCP) (25). This data has 1.05 mm isotropic resolution and approximately 65 diffusion weighting directions spread over two shells with b-values of 1,000 and 2000 s/mm^2^. For each of the 178 subjects in the HCP dataset, a nonlinear (i.e., deformable) transform mapping from the MNI152 nonlinear 2009c brain (59) to that subject’s brain was found using image registration tools from the ANTs software package (26) after brain extraction. Each patient’s transform was used to warp the VIM region of interest (ROI) from the DISTAL Medium atlas (27), and the precentral gyrus ROI from the Harvard-Oxford atlas (28–31), from MNI space to the subject’s brain to serve as the seed and terminus regions, respectively, for tractography. Probabilistic tractography from VIM to the precentral gyrus (VIM-precentral) (Figure 1a) was performed using FSL software (60). FSL bedpostx determines the distribution of diffusion parameters at each voxel, automatically determining the number of and modeling crossing fibers. The subject’s network-based VIM-precentral hotspot was created as follows: for each voxel within the subject-fit VIM ROI, the intensity of the VIM-precentral hotspot is the percent of streamlines launched from that voxel that reached the ipsilateral precentral gyrus ROI (determined using FSL probtrackx2’s “--os2t” output seeds to terminus option). The inverse of the MNI-to-subject transform was applied to the subject’s network-based hotspots to warp them back to MNI space. Finally, the 178 MNI-space network-based VIM-precentral hotspots (each specific to one of the 178 HCP subjects) were median-averaged to create normative, network-based VIM-precentral hotspot objects. The normative VIM-precentral hotspot object was divided by its maximum values to form the VIM-precentral regions in the NTA, which thus ranges from 0 to 1. Note that for the NTA we are using VIM-precentral to refer to the normative representation of the seeds to termini, not the tract itself. These NTA regions (Figure 1b) are shared at https://github.com/adatta92/VIM2precentral.

### Patient-fitting of NTA regions and calculation of normative tractographic coefficients

After FSL brain extraction of both the MNI152 and patient preoperative T1 images, a nonlinear transform mapping from the MNI brain to each patient’s brain was found using ANTs image registration tools. The inverse of this transform was used to warp the NTA VIM-precentral objects to each patient’s T1-space. A rigid transform between the patient’s postoperative (either T2-weighted-CUBE or FGATIR) MRI and preoperative T1-weighted MRI was also found using ANTs (no brain extraction). The manually segmented FUS thalamotomy lesions (as described in the “lesion segmentation” subsection above) were coregistered to the patient-fit NTA hotspots using this transform. To quantify the degree to which the lesion falls within the patient-fit NTA VIM-precentral regions, we calculated the average value of the patient-fit NTA object over the voxels of the coregistered lesion segmentation. This quantity was named the normative tractography coefficient (NTC). A lesion that only contained the voxel where the patient-fit NTA hotspot is at its maximum would thus have an NTC of 1, while a lesion that does not overlap with any of the patient-fit NTA object would have an NTC of 0. In practice, none of our NTC values reached either of these extremes.

### Use of standard clinically acquired DTI for probabilistic tractography

The clinically acquired (lower resolution) DTIs (3 T, 1 mm x 1 mm x 2 mm resolution, 30 diffusion weighting directions, b-value of 1,000 s/mm^2^), were used to run patient-specific probabilistic tractography using FSL. Tracking was performed from the patient-fit VIM to the patient-fit precentral gyrus, as done in the 7 T HCP datasets. Dentatorubrothalamic probabilistic fiber tracking was also attempted from the thalamus to the hand knob region of the motor cortex to mirror the probabilistic tractography performed with high-resolution DTIs in (32). We tabulated the number of patients in whom probabilistic tracking from the VIM to the precentral gyrus was successful.

## Results

### Tremor suppression and relationship to patient-reported QoL outcome

**Figure 2:**
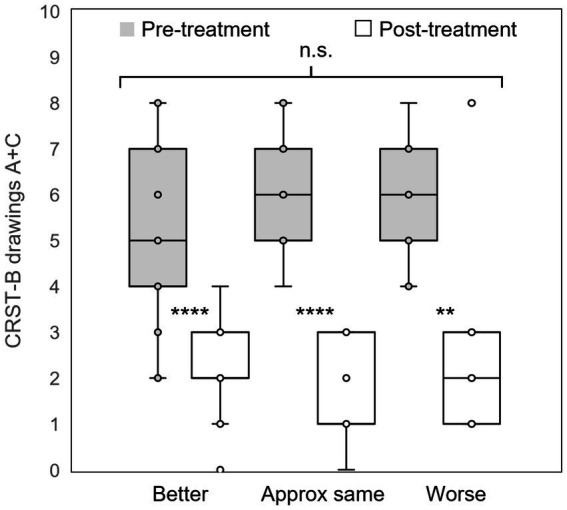
Immediate post-procedural tremor reduction. Groups of patients who self-assessed their quality of life as “better,” “approximately the same,” or “worse” at last follow-up all experienced significant reduction tremor as measured by the CRST part B Archimedes spiral and straight-line drawings on the day of treatment; however, there was no difference in tremor reduction between groups. CRST: Clinical Rating Scale for Tremor; **: *p* < 0.01; ****: *p* < 0.001; n.s.: not significant.

**Figure 3:**
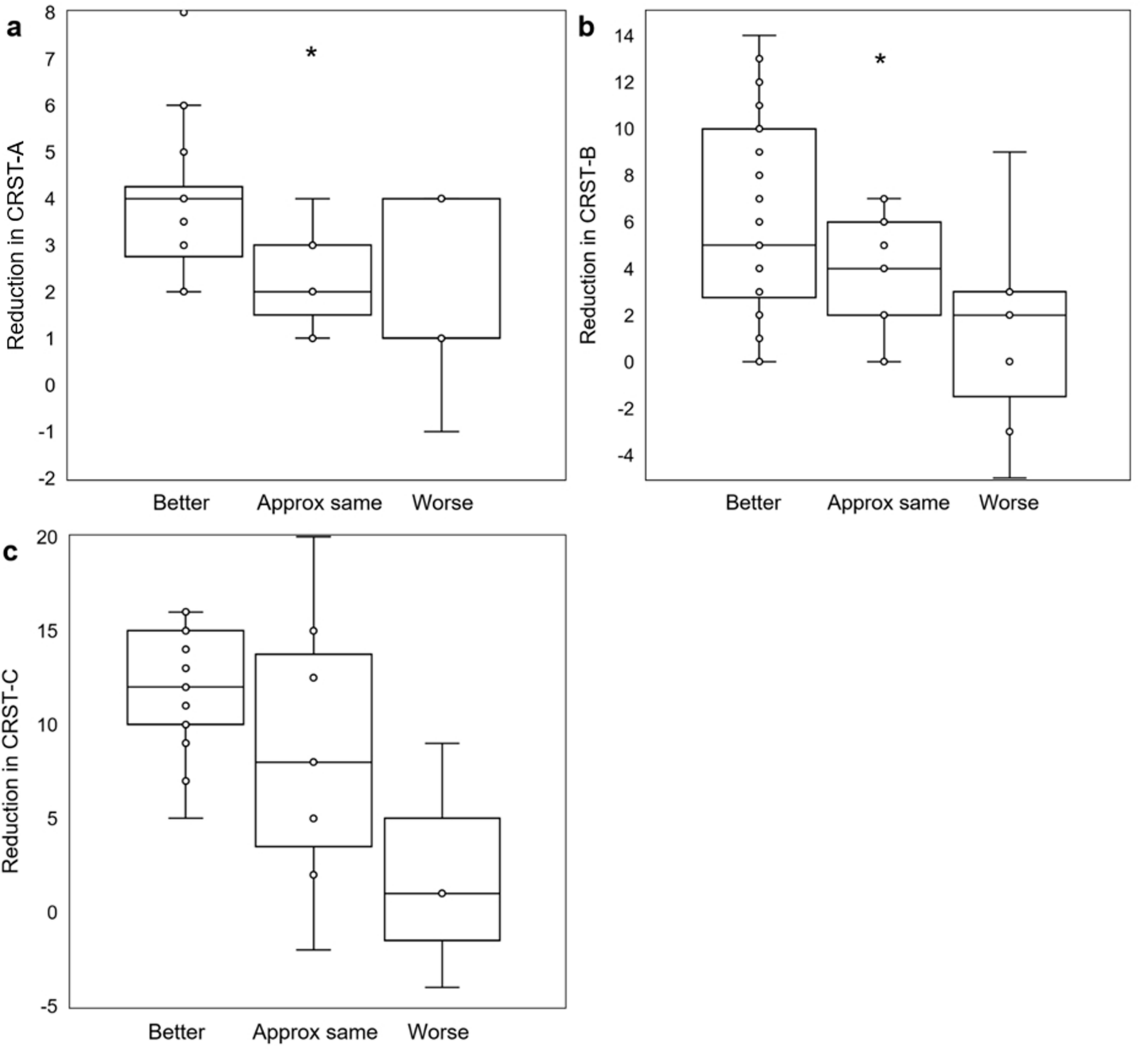
Objective post-MRgFUS tremor reduction and association with QoL self-assessment. Box and whisker plots demonstrate significant associations (by Kruskal-Wallis test) between objective measures of tremor reduction (a: CRST part A, treated hand only; b: CRST part B, treated hand only, without pouring; c: CRST part C, function only) and QoL outcomes. CRST: Clinical Rating Scale for Tremor; *: *p* < 0.05.

Sixty patients (76.0 ± 1.10 years) reported their self-assessed change in QoL at last follow-up (405 ± 44 days) post-treatment. Of those, 37 (61.7%) rated their QoL as “better,” 14 (23.3%) rated their QoL as “approximately the same,” and 9 (15%) rated their QoL as “worse” since treatment. On the day of each patient’s MRgFUS procedure, scores from CRST part B drawings sections A and C were calculated immediately before and after treatment. Each group of patients stratified by patient-reported QoL assessment at last follow-up had significant reduction in tremor on the day of treatment (QoL “better”: CRST-B section A + C 5.5 ± 0.31 to 2.1 ± 0.17; QoL “approximately the same”: 6.2 ± 0.36 to 1.7 ± 0.29; QoL “worse”: 6.0 ± 0.47 to 2.6 ± 0.73; all *p* = 0.009), with similar tremor reduction across all groups (Kruskal-Wallis *H* = 3.5697, *p* = 0.168; Figure 2). Thus, immediate post-procedure tremor reduction did not vary between categories of patient-reported change in QoL at last follow-up. At last follow-up, 31/32 (96.9%), 36/44 (81.8%), and 21/23 (91.3%) patients had improved scores on CRST parts A (in the treated hand only), B (treated hand only without pouring), and C (function only, not including global assessment), respectively. Five of the part A, four part B, and two part C scores were measured at less than 90 days after the patient’s procedure, but all were at greater than 30 days post-procedure. There was a significant association between level of tremor reduction at last follow-up as measured by CRST subpart scores and QoL category (part A: *H* = 6.6039, *p* = 0.036; part B: *H* = 6.5706, *p* = 0.037; part C: not enough respondents; Figures 3a–c).

**Figure 4:**
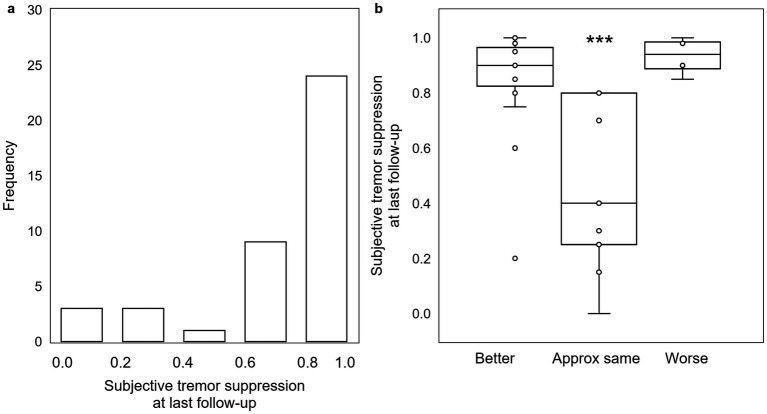
Subjective assessment of tremor reduction at last follow-up does not follow the expected pattern. (a) While the majority of patients experienced between 80% and 100% subjective tremor suppression, (b) there is no significant difference in degree of subjective tremor suppression between patients self-assessing as “worse” and those self-assessing as “better.” Those that self-assess as “approximately the same” had a significantly lower degree of subjective tremor suppression. ***: *p* < 0.001.

At last follow-up, patients reported an average subjective tremor suppression of 78.4 ± 4.1%, with the majority reporting ≥80% reduction (Figure 4a). While there is significant interaction between subjective tremor suppression magnitude and QoL category (*H* = 15.923, *p* = 0.0003; as there were only four reported tremor suppression scores in the “worse” QoL group, an additional value equivalent to the mean of the four “worse” QoL tremor suppression scores was added to the “worse” QoL group in order to obtain the requisite five values necessary for Kruskal-Wallis testing), there is no significant difference between the subjective tremor reduction for patients who rated their QoL “better” versus for those who rated their QoL “worse” (*p* = 0.44; Figure 4b). Both groups reported relatively high subjective tremor suppression (“better”: 86.8 ± 3.1% versus “worse”: 93.2 ± 3.5%), whereas the group rating their subjective QoL as “approximately the same” by last follow-up reported less subjective tremor reduction (46.7 ± 10.4%).

### Adverse effects and QoL outcomes

**Figure 5:**
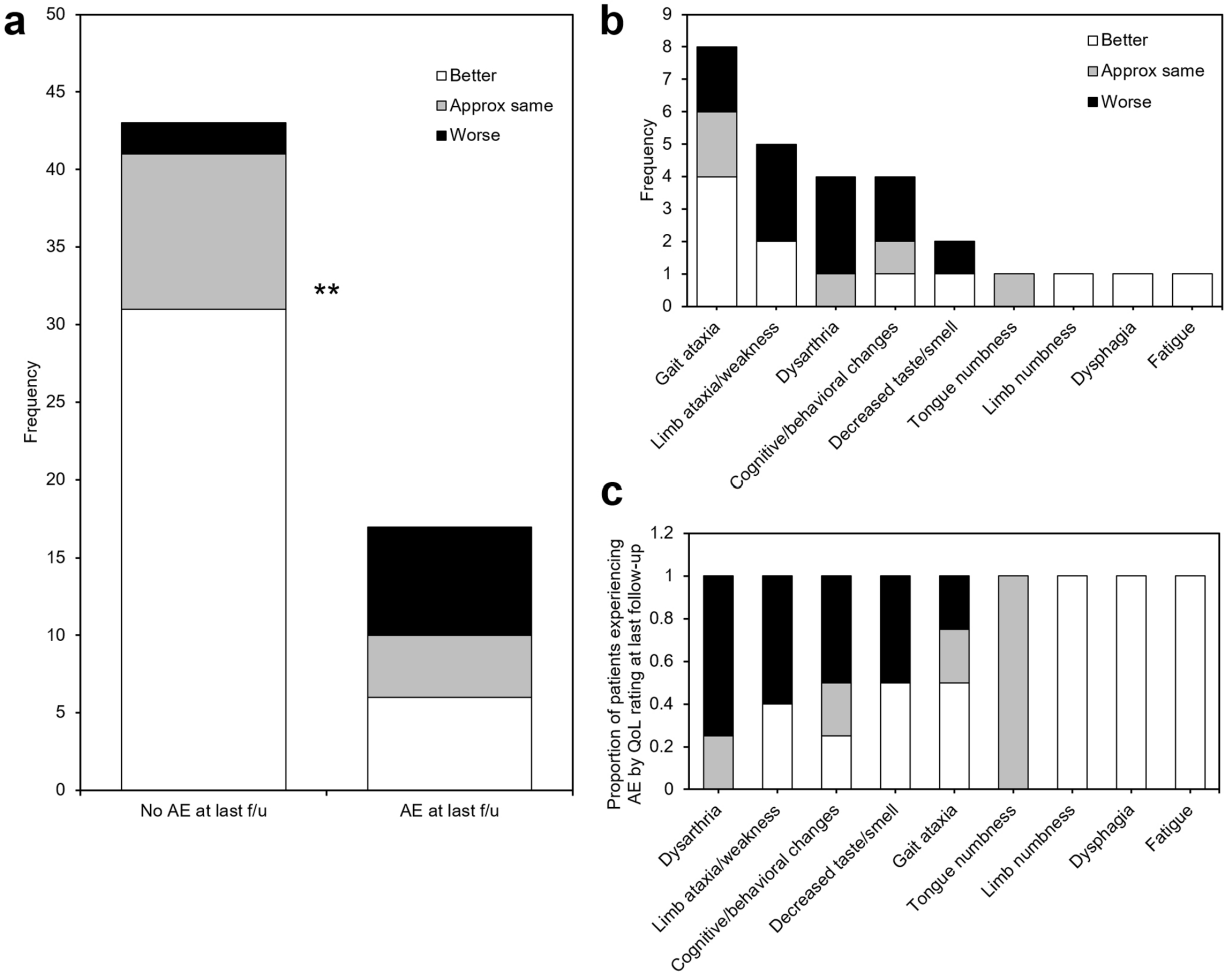
Adverse effects after MRgFUS VIM thalamotomy. (a) histogram of adverse effects experienced by patients at last follow-up, stratified by study-end QoL self-assessment. (b) A significantly larger proportion of patients who reported unchanged or worse QoL experienced persistent adverse effects at last follow-up. (c) Adverse events ranked in order of proportion of patients experiencing that AE reporting worse (black), same (gray), or better (white) QoL at last follow-up. AE: adverse event; f/u: follow-up; **: *p* < 0.01.

Most patients (50/60, 83.3%) experienced some adverse effect (AE) at the time of the first follow-up phone call (4.89 ± 1.35 days). All patients in this cohort received intraoperative steroids and a standard, postoperative one-week steroid taper. Thirteen patients (21.6%) had persistent self-reported sensorimotor AEs at the time of last follow-up. The most frequent sensorimotor AE experienced by patients was gait ataxia (8/60, 13.3%), followed by contralateral limb ataxia or weakness (5/60, 8.3%), dysarthria (4/60, 8.3%), and decreased sense of taste or smell (2/60, 3.3%); one patient each (1.7%) experienced tongue numbness, contralateral limb numbness, dysphagia, or fatigue (Figure 5a). Additionally, four patients self-reported cognitive or behavioral changes after the procedure (6.7%). The AEs with the highest proportion of patients experiencing that AE who reported “worse” QoL at last follow-up were dysarthria (3/4, 75%), limb ataxia/weakness (3/5, 60%), cognitive/behavioral changes (2/4, 50%), decreased taste/smell (1/2, 50%), and gait ataxia (2/8, 25%) (Figure 5c). Additionally, no patients who experienced dysarthria at last follow-up rated their QoL as “better” than before the procedure.

### Effect of skull density ratio, dose volume, lesion size, and normative tractographic coefficients on QoL outcomes

Stratified by patient QoL assessment at last follow-up, the skull density ratio (SDR) of “worse” patients was highest (0.64 ± 0.03), followed by “better” (0.58 ± 0.02), and lastly “same” (0.53 ± 0.03), with the group differences trending toward significance (Kruskal-Wallis H = 4.7705, *p* = 0.09). For most patients, SDRs were calculated from preoperative CT scans acquired using a GE Revolution CT at our institution. For any patient’s imaged elsewhere using non-GE CT scanners, a correction factor is utilized by Insightec to attempt to normalize SDRs to the GE-derived standard.

**Figure 6:**
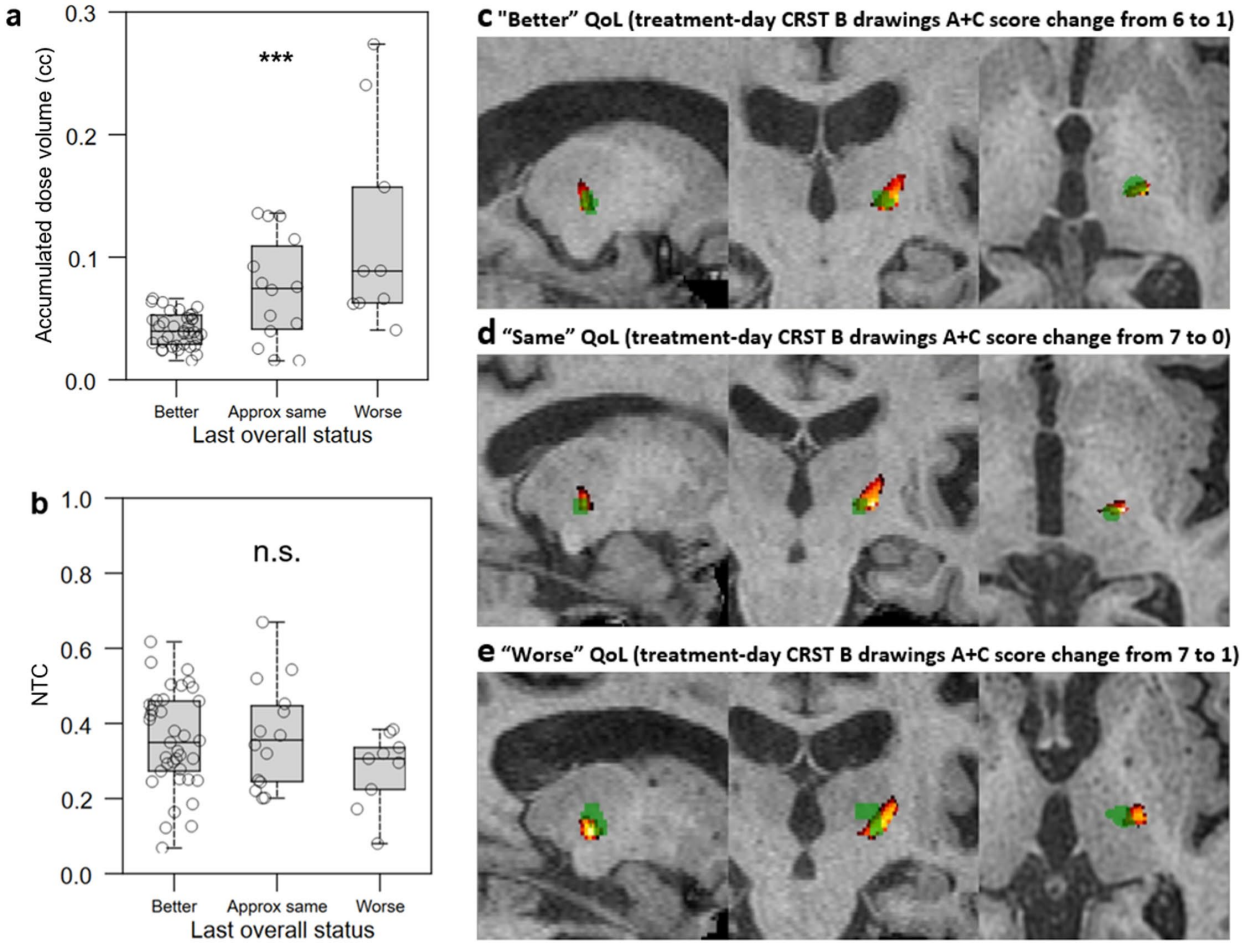
Relationship between lesional characteristics and QoL outcomes. (a,b) Lower accumulated dose volume (a) but not greater normative tractographic coefficient (NTC) between the lesion and VIM-precentral NTA hotspot (b) was significantly associated with difference between all three subjective QoL assessment groups. c-e: FUS thalamotomy lesions (green) and NTA hotspots (hot colors, thresholded to >0.6 to minimize the black/empty VIM component) for exemplar patients who self-assessed their QoL at last follow-up to be “better” (c), “approximately the same” (d), and “worse” (e); despite having similar treatment-day reductions in tremor, the lesion of the patient whose QoL improved was smaller and best encapsulated by the NTA hotspot, while that of the patient whose QoL decreased was largest, with much of the ablated volume superior and medial to the hotspot. The “same” QoL patient had the smallest lesion of the three, and it is slightly medial to the brightest voxels of the hotpot. NTC: normative tractography coefficient for VIM-precentral hotspot; QoL: quality of life; CRST: Clinical Rating Scale for Tremor; ***: *p* < 0.001; n.s.: not significant.

Lesion characteristics were calculated and stratified by patient QoL assessment at last follow-up (Figures 6a,b). Smaller accumulated dose volume (Kruskal-Wallis *H* = 14.2693, *p* = 0.0008; Figure 6a) was significantly associated with greater subjective QoL assessment. FUS thalamotomy lesions (green) and NTA hotspots thresholded to >0.6 (hot colors) are shown for exemplar patients who self-assessed their QoL at last follow-up to be “better” (Figure 6c), “approximately the same” (Figure 6d), and “worse” (Figure 6e). Despite having similar treatment-day reductions in tremor, the lesion of the patient whose QoL improved was smaller and best encapsulated by the NTA hotspot, while the lesion of the patient whose QoL decreased was largest, with much of the ablated volume superior and medial to the hotspot. The “same” QoL patient had the smallest lesion of the three, located slightly medial to the brightest voxels of the hotpot.

**Figure 7:**
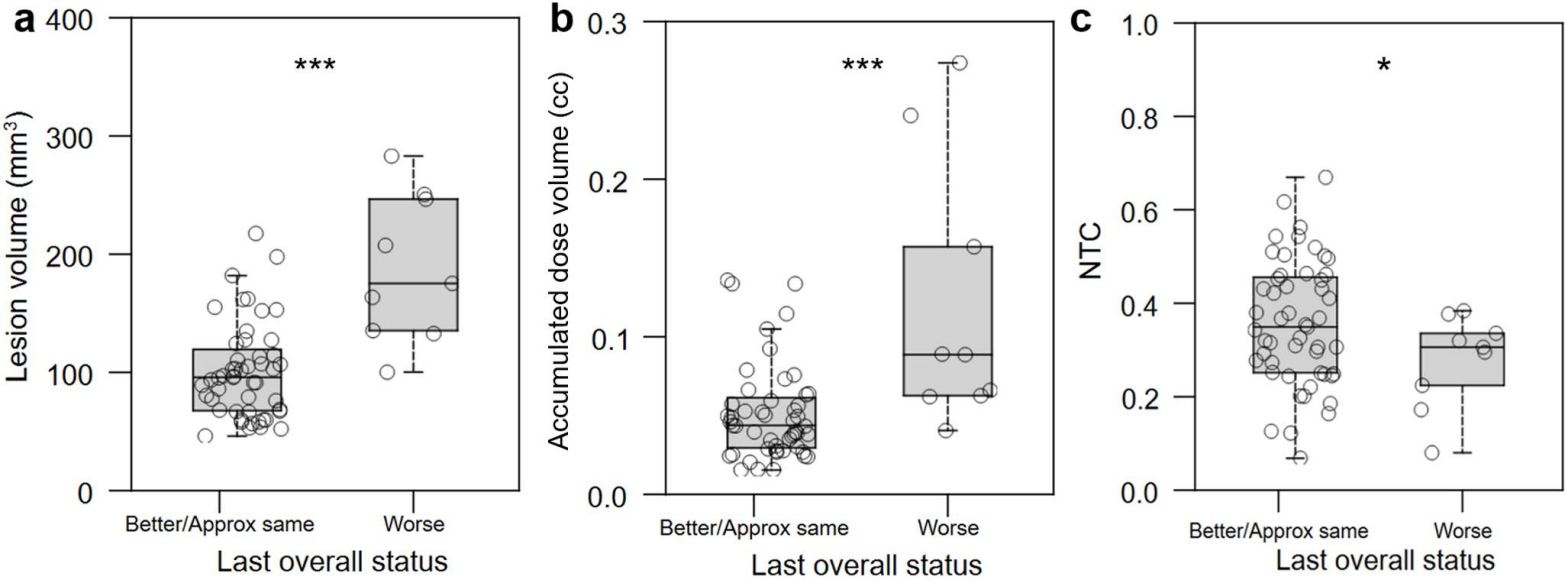
Relationship between lesional characteristics or normative tractography coefficient and binary QoL outcome. (a,b) Better/same QoL outcomes were significantly associated with smaller lesion volume (in mm^3^) and accumulated dose volume (in cc). (c) Improved or unchanged QoL was significantly associated with normative tractographic coefficient (NTC) with the NTA VIM-precentral hotspot. *: *p* < 0.05; ***: *p* < 0.001.

To facilitate calculation of classifiers that would allow us to avoid worse QoL outcomes, we binarized QoL outcomes by combining patients who self-assessed as “better” and “approximately the same” into a single “better/same” group. “Better/same” QoL outcomes were significantly associated with smaller lesion volume (*p* < 0.0001; Figure 7a), smaller accumulated dose volume (*p* < 0.0001; Figure 7b), and higher normative tractographic coefficient with the NTA VIM-precentral hotspot (NTC; *p* = 0.046; Figure 7c).

To determine values of intraprocedural treatment parameters and of normative tractographic coefficients that might optimize for better QoL outcomes, receiver operating characteristic curves were generated and optimization points with maximum Youden index were identified that maximize the balance between sensitivity and specificity (Table 2). Immediate post-operative lesion volume less than 127 mm^3^, intraprocedural accumulated dose volume less than 60 mm^3^, and lesion/VIM-precentral coefficient (NTC) greater than 0.54 were all associated with “better/same,” versus “worse,” QoL outcome.

**Table 2:** Test performance of lesional characteristics as univariate predictors of QoL outcomes.

Variable	AUC	Youden Index	Sensitivity	Specificity	Optimal Cutoff	*p*
Lesion volume (mm^3^)	0.880	0.692	0.804	0.889	<127.4	<0.0001
ADV (cc)	0.826	0.634	0.745	0.889	<0.0594	<0.0001
NTC [0–1]	0.723	0.438	0.549	0.889	>0.544	0.015

AUC: area under curve; ADV: accumulated dose volume; NTC: normative tractography coefficient for VIM-precentral hotspot. Receiver operating curves were generated for lesion volume and degree to which the lesion is within the NTA VIM-precentral projection-based hotspot. Optimization by setting a threshold at maximum Youden index to maximize the balance between sensitivity and specificity yielded optimal cutoff values for lesion characteristics.

Since late 2018, the stereotactic coordinate that was used for targeting has been 11 mm from the lateral wall of the third ventricle, ¼ of the anterior commissure-posterior commissure (AC-PC) distance anterior to PC, and 2 mm above the intercommissural plane, similar to (33). There is no association between the Euclidean distance from the stereotactic coordinate to the center of mass of the lesion and QoL status (Kruskal-Wallis H = 1.4206, *p* = 0.49).

### Relationship between lesion volume and self-reported and clinically rated tremor suppression

**Figure 8:**
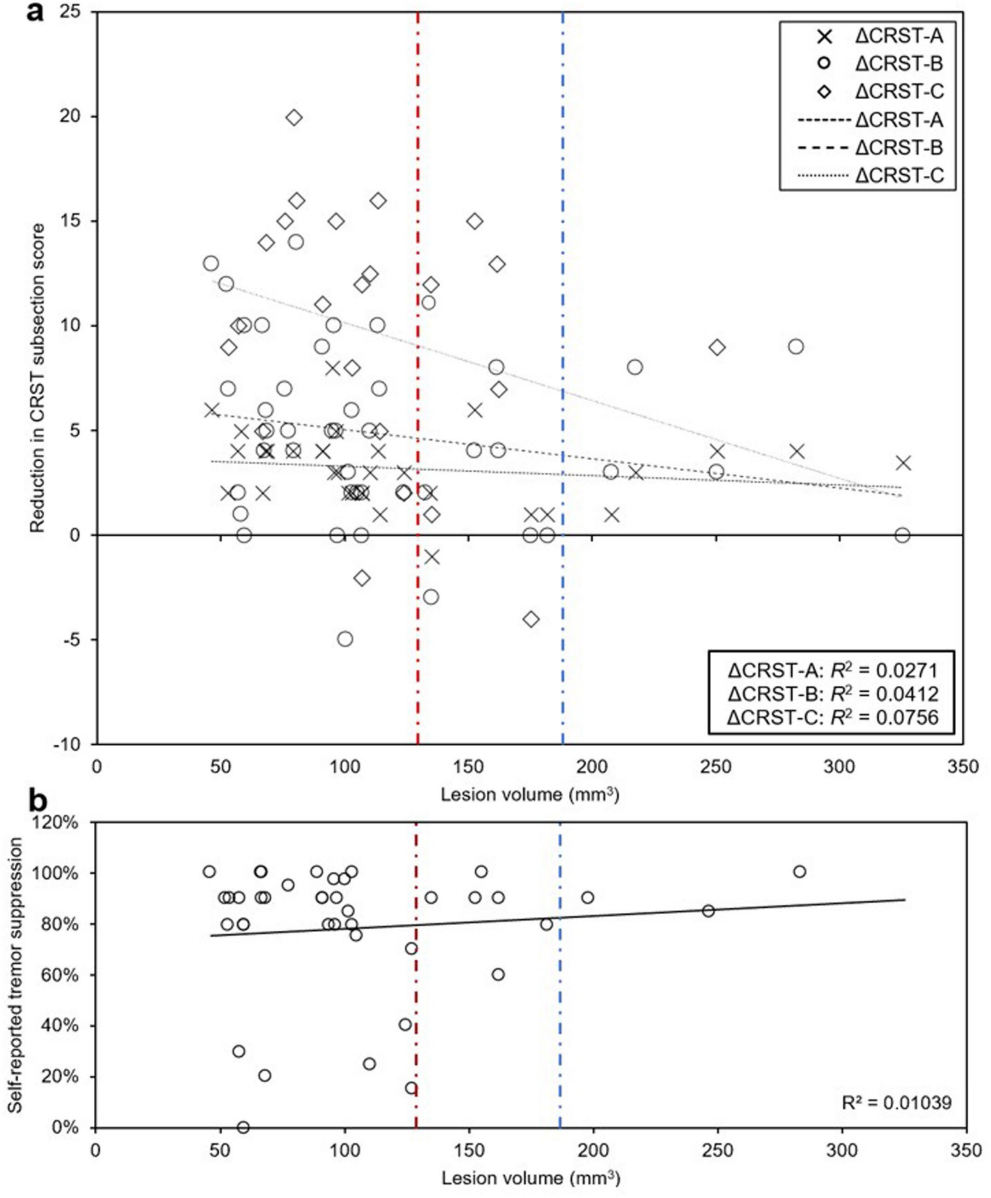
Relationship between lesion size and clinically rated and self-reported tremor suppression. (a, b) There were no significant associations between lesion volume and CRST part A (treated hand only), part B (treated hand only, no pouring), part C (functional assessment only) (a), or self-reported tremor suppression (b) at last follow-up. Above lesion volume of 180 mm^3^, no patients had lower than expected subjective or objective tremor suppression at last follow-up (blue dashed line). All patients that had less than 50% subjective tremor suppression had lesion volumes < 127.4 mm^3^ (red dashed line), which represents the lesion volume threshold below which patients were more likely to have “better” or “same” QoL.

In the setting of our finding that smaller lesions (less than 127 mm^3^) were associated with better QoL outcomes, we next examined the relationship between lesion size and both objective and subjective measures of tremor suppression. There was no significant association between lesion size and changes in CRST part A (treated hand only), part B (treated hand only without pouring), or part C (function only) (*R*^2^ = 0.0123, *R*^2^ = 0.0271, *R*^2^ = 0.0412, and *R*^2^ = 0.0756, respectively; Figure 8a), or between lesion size and self-reported tremor suppression at last follow-up (*R*^2^ = 0.01039; Figure 8b). With lesions greater than 180 mm^3^, all patients reported effective subjective tremor control at last follow-up and objectively scored at or above the predicted tremor reduction trendline on CRST subscales, but they were more likely to report “worse” QoL.

### Effect of procedural characteristics on lesion volume

No direct correlation was found between the number of sonications with temperature above 50°C and lesion volume, nor between the total energy applied and lesion volume.

### Use of clinically acquired low-resolution DTI for creation of probabilistic dentatorubrothalamic tracts

Using the clinically acquired low-resolution DTIs (3 T, 1 mm x 1 mm x 2 mm resolution, 30 diffusion weighting directions, b-value of 1,000 s/mm^2^), probabilistic tractography from the VIM to the precentral gyrus using FSL was successful in only 44 of the 60 patients in this cohort. In 11 patients, the format of the “blip-down” acquisition used for artifact correction before fiber tracking precluded use. In another five cases, the DTIs had too much artifact for reasonable fiber tracking. However, even in the 44 patients with adequate DTIs, specialized tracking to the hand-knob subregion of precentral gyrus, which was found by (32) to be the most predictive DRTT methodology, was unsuccessful in all patients.

### Effect of lesion size and normative tractographic coefficient on adverse effects

**Figure 9:**
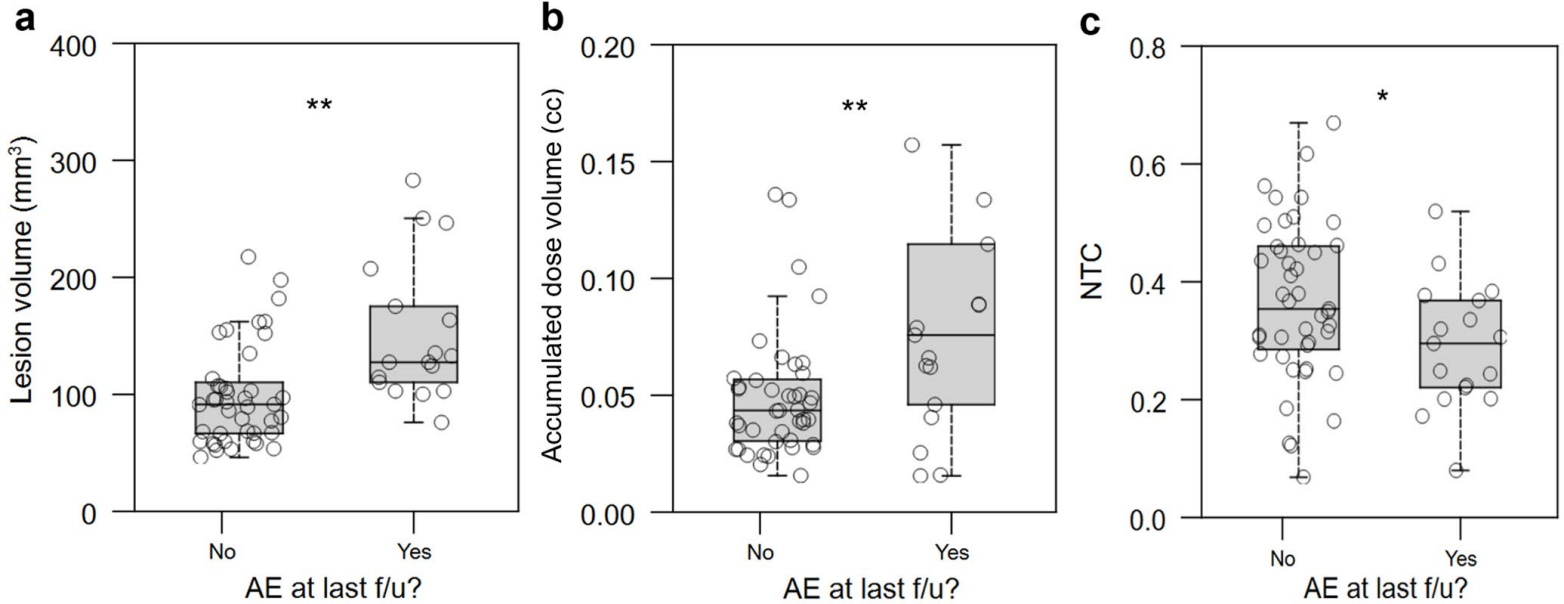
Relationship between lesional characteristics and presence of persistent AEs at last follow-up. Larger lesion volume, (a) accumulated dose volume (in cc) (b), and normative tractographic coefficient (c, NTC) with the VIM-precentral projection-based hotspot in the NTA were significantly associated with the presence of persistent AEs at last follow-up. AE: adverse effect; NTC: normative tractography coefficient. *: *p* < 0.05; **: *p* < 0.01; n.s.: not significant.

We examined the relationship between lesion characteristics and the presence of AEs at time of last follow-up. Larger lesion volume, larger accumulated dose volume, and lower NTC were associated with presence of AEs at last follow-up (*p =* 0.002, Figure 9a; *p =* 0.002, Figure 9b; *p* = 0.020, Figure 9c, respectively).

## Discussion

In this retrospective study, we sought to investigate the relationship between subjective QoL outcome and tremor suppression (both subjectively reported and clinically measured), and to determine the feasibility of a scalable approach to MRgFUS thalamotomy lesioning that could potentially optimize QoL outcome. We focused on subjective, qualitative, patient-reported QoL instead of the Quality of Life in Essential Tremor (QUEST) questionnaire (16) because QUEST frames QoL chiefly in terms of the impact of tremor, while we hypothesized that the degree of tremor suppression is not necessarily entirely predictive of post-treatment QoL, with the impact of adverse side effects also playing a role.

### What is the relationship between subjective QoL outcome and tremor suppression?

On the day of MRgFUS thalamotomy, all patients tested immediately post-procedure had improved tremor scores, regardless of QoL outcome at last follow-up. For the majority of patients, improvement was durable through last follow-up. At last follow-up, objective, quantitative tremor suppression correlated with QoL outcome (Figure 3); this is unsurprising, as a patient without significant objective improvement in tremor is unlikely to rate their QoL as “better” after their procedure. However, patients with *either* “better” or “worse” QoL after treatment *perceived* similarly high levels of subjective tremor improvement (approximately 80–100%), while patients who rated their QoL as “approximately the same” *perceived* an average of only approximately 50% (Figure 4b). In other words, a group of patients who *perceived* strong improvement in tremor nevertheless rated their QoL as “worse” after treatment. We hypothesized that the distinguishing factor between these patients and others is the set of adverse effects (AEs) they experienced; indeed, 77.8% (7/9) of patients reporting “worse” QoL (with or without tremor improvement at last follow-up) had persistent AEs at last follow-up, compared to just 16% (6/37) of patients stating “better” QoL (*p* = 0.001; Figure 5). Of the two” worse” patients without AEs at last follow-up, one had worsened tremor relative to before procedure as quantified by CRST parts A (treated hand only) and B (treated hand only, without pouring). The other was later diagnosed with normal pressure hydrocephalus, so the cause of their worsened QoL could be multifactorial. The relative impact of AEs versus tremor control on a patient’s self-assessed QoL rating may depend on the type and severity of their persistent AEs, as well as the effect that their AEs have on their lifestyle. Interestingly, even though gait ataxia was the most common, dysarthria and limb ataxia/weakness were the most likely AE’s leading to “worse” QoL assessments, while gait ataxia was better tolerated (Figure 5). Overall, though a high degree of subjective tremor suppression appears necessary for achieving the highest QoL outcome, it is not sufficient, and minimizing AEs may be required to promote higher QoL ratings.

How can AEs be avoided during FUS procedures? Larger lesions are thought to contribute to a higher side effect profile (33, 34). We identified that large lesion volume strongly differentiated patients with persistent AEs at last follow-up (Figure 9a) as well as the “worse” QoL outcome group (Figure 7a). However, given that final lesion size may continue to develop for days after treatment, a proxy quantity is needed that can be measured and monitored in real time during treatment and that also correlates with AE frequency and QoL. Accumulated dose volume is an intraprocedural metric that is strongly correlated with postoperative lesion volume (35). Both larger lesion size measured on immediate post-treatment MRI (Figure 7a) and higher intraprocedural accumulated dose volume (Figure 7b) strongly distinguished worse QoL outcome. Cutoff values were found, with lesion size above 127 mm^3^ and accumulated dose volume of greater than 0.06 cc (60 mm^3^) strongly predicting “worse” QoL outcome (Table 2).

However, we also found that patients with lesions larger than approximately 180 mm^3^ uniformly had both subjective and objective improvement in tremor, while some patients with lesions smaller than 180 mm^3^ had less effective tremor control (Figure 8). This value contrasts with 127 mm^3^, the lesion size below which patients are more likely to have “better/same” than “worse” QoL outcomes (Table 2). At first glance, these data are contradictory – why do lesions larger than 180 mm^3^ predict the best subjective and objective tremor suppression, while lesions *smaller* than 127 mm^3^ predict better QoL? We hypothesized that even more important for predicting QoL than the size of the lesion is the precise location of the lesion and the clinical consequences thereof. Presumably, a well-placed lesion under 127 mm^3^ in size will target the putative “sweet spot” that optimizes for tremor control, improves QoL, and minimizes AEs; a lesion larger than this might improve tremor but cause AEs and therefore diminish QoL, while a smaller lesion that misses the “sweet spot” may not be large enough to improve tremor, even if it spares the patient of AEs.

### Is there a scalable approach to MRgFUS thalamotomy lesioning that can optimize QoL outcome?

Central to improving patient satisfaction with MRgFUS thalamotomy is devising an approach that can be used to model the small target zone within each patient’s imaging space that will lead to a superior QoL outcome (which incorporates maximizing subjective and objective tremor suppression). Distance between the stereotactic coordinate and lesion center-of-mass was not associated with QoL outcome, suggesting that canonical targeting does not provide this. Though studies have highlighted the utility of personalized DTI in predicting putative ablation zones within VIM (19, 20, 36, 37), the acquisition and processing of high-resolution personalized DTI may be costly, technically challenging, and resource-intensive. As such, the ability to scale across centers may be limited, particularly in more community-based settings. Lower resolution DTI acquisition may be more achievable due to the decreased time and resources required, but may lack reliability. Using our clinically acquired (lower resolution) DTIs (3 T, 1 mm x 1 mm x 2 mm resolution, 30 diffusion weighting directions, b-value of 1,000 s/mm^2^), we were only able to successfully run probabilistic tractography on less than 3/4 of the patients in this cohort. Since patients with ET often also have head tremor, we presume that acquiring reliable DTI, which is extremely susceptible to motion artifact, may be more challenging in this patient population than in the general population. Further, even in the patients with adequate DTIs, specialized tracking to the hand-knob subregion of precentral gyrus, as done with the higher resolution research DTIs in (32), and found to be the most reliable methodology for generating outcome-predictive streamlines, was unsuccessful in all patients in this cohort. This may be due to the non-isotropic resolution, inferior angular resolution (lower number of diffusion weighting directions), and lower b-value of the clinical-grade DTIs, which are not optimal for tractography. Together, these challenges highlight the need for a scalable, resource-light approach utilizing high-resolution DTIs.

Here, we propose the use of a large-sample-size, high-resolution DTI database from the Human Connectome Project to create a normative tractographic atlas (NTA) to provide representations of VIM subregions with high probability of streamlines to the motor cortex that are then fit to each patient. This approach is directly scalable as it does not require any DTI acquisition, can be performed using the standard volumetric preoperative T1 imaging, and only requires freely available coregistration algorithms to MNI space. Our data demonstrate that smaller lesions (measured both intraprocedurally with accumulated dose volume and at time of immediate postoperative imaging with lesion segmentation) and higher NTC independently predict superior QoL outcomes. Furthermore, our analyses yield a set of threshold values for immediate postoperative lesion size (< 127.4 mm^3^), accumulated dose volume (< 0.06 cc) and NTC (> 0.54) that select against worse QoL outcomes and that could one day be used prospectively, at time of treatment, to plan a lesion that maximizes the chance of improving overall QoL by optimizing the tradeoff between maximum tremor suppression and minimum AEs.

Strategies that are now used at our institution to limit lesion size include the use of fewer total sonications, increasing power instead of duration to reach the desired sonication energy, targeting a lower peak temperature of about 55°C rather than 57-60°C, and the application of masks that deactivate elements primarily transmitting through the temporal bones, thereby limiting medial-lateral spread of the thermal spot. Fewer sonications are achieved by aligning with minimal power and then rapidly ramping the power to treatment power levels. Prior to July 2020, when the patients in this study were treated, only the application of masks was routinely used. In addition, in this cohort, we did not find a direct correlation between the number of sonications with temperature above 50°C and lesion volume, nor between the total energy applied and lesion volume. The lack of relationship between total energy applied or number of sonications and lesion volume may be a result of the numerous differences between patients and their treatment parameters that are challenging to control for, including the number, magnitude, and direction of target adjustments, as well as SDR distribution over the skull.

Many centers offering MRgFUS for ET acquire postoperative imaging approximately 24 h after treatment. We expect that the segmented regions (zones I and II) grow during the first 24 h postop, so the lesion volumes in this study may not be directly comparable to those stated in studies from other institutions. In addition, note that the NTC is not a similarity metric – our hypothesis was that an adequate portion of the patient-fit NTA object needs to be ablated, not its entirety. Too large of a lesion may increase the likelihood of adverse side effects, and too small of a lesion may lead to suboptimal subjective tremor suppression.

Within the range of last follow-ups for which we have data, patients with longer follow-up did not have less tremor improvement at last follow-up, even when considering only subjects with smaller lesions (Supplementary Figure S2). This suggests that most patients, including those with smaller lesions, did not have tremor recurrence during the study period, but future work looking at longer follow-up (e.g., > 5 years) is merited.

Finally, it is important to note that though the desired ablation volume may coincide strongly with the NTA network-based hotspot, we do not recommend targeting at the center of mass of the NTA object as this is likely to result in ablative ultrasonic dose in the internal capsule. Due to the predominant inferoanteriolateral spread of ultrasonic energy during most MRgFUS procedures, our current practice is to target at the medial apex of the NTA (typically about 15 mm lateral to midcommissural plane, 1 mm anterior to the 25% ACPC distance from PC, and 2 mm superior, Figure 1b). This is approximately 3.5 mm away from the thalamocapsular border at 2 mm above ACPC, and approximately 2.5 mm away from the thalamocapsular border at ACPC. The thalamocapsular border is best seen on FGATIR imaging, though it can also be seen on T1-weighted imaging with appropriate contrast windowing. The exact location and relative ACPC coordinates of this medial apex varies on an individual patient basis due to the nonlinear coregistration from MNI space to each patient’s native T1 space.

### Limitations and future directions

The primary limitations of our study include the relatively low number of patients available for purely unbiased retrospective analysis (patients treated prior to the creation of the NTA hotspot pipeline at our center), and incomplete data due to lack of consistent follow-up in this cohort. The relatively short follow-up period (405 ± 44 days) limits our ability to assess the durability of tremor improvement (or the likelihood of AEs resolving) in the long run, and how this impacts QoL.

Another limitation of the proposed NTA method is that the HCP 7 T datasets used to determine the normative VIM-to-precentral hotspots were acquired from healthy adults. While the nonlinear registration used to warp the NTA from the MNI brain to each patient’s T1-weighted MRI has some capacity to account for differences in ventricular size and morpohology commonly seen with aging, it is possible that our proposed method would be less reliable if a patient has other pathology, such as tumor, large stroke, encephalomalacia, or other structural abnormalities. In such cases, patient-specific tractography may likely be an important adjunct.

Future directions could include obtaining both subjective, qualitative, patient-reported QoL data and QUEST data in patients to compare our simplified scale, which is intended to include the impact of both tremor reduction and any adverse side effects, to QUEST, which focuses on the effect of tremor on specific ADLs and functional aspects. Qualitative data could also probe more subjective insights from patients based on their experience including whether or not they would have decided to undergo MRgFUS in the first place, or why they would or would not undergo MRgFUS in the future for the second side. Additionally, future work might include refining the NTA by focusing on only the most efficacious components of the VIM to precentral fibers in order to get a tighter normative hotspot. Tracking to the hand-knob region instead of all of the motor cortex could possibly accomplish this (as shown in (32)). This would be aided by an effective method for automatically segmenting the hand-knob region in the HCP dataset subjects and is thus outside of the scope of this manuscript. Finally, in this study, intraoperative imaging used the body RF coil; future work could include using a 2-channel head coil designed to be compatible with the transcranial MRgFUS setup, which has been shown to enable better visualization of the thalamus and other structures, and more precise MR thermometry (38).

Our data nevertheless suggest that this relatively simple approach can be used to optimize patient QoL and satisfaction with MRgFUS thalamotomy. Future work to validate our findings prospectively and to automate the computational aspects of our approach will be important to facilitate wider adoption of this approach.

## Conclusion

Though tremor suppression is certainly required for achieving the best QoL outcome after MRgFUS thalamotomy for ET, paying particular attention to minimizing adverse effects may be more impactful to QoL than the exact degree of tremor suppression achieved. We find that small lesions (both as predicted by accumulated dose volume at time of treatment and on postoperative imaging) that fall within the sweet spot of our NTA may provide this optimal balance. Of particular interest, we find a cutoff value of intraprocedural accumulated dose volume < 0.06 cc as optimal to avoid poor QoL outcome. Furthermore, since generating the NTA volume requires only routine, preoperative T1-weighted imaging and is not dependent on resource-intensive high-resolution DTI acquisition, the NTA represents a reliable and easily scalable method that can be implemented anywhere MRgFUS is performed. Eventually, such reliable and scalable image-guided targeting techniques, in addition to patient-specific modeling when available, with predictive intraprocedural metrics such as accumulated dose volume, may obviate the current need for an awake, interactive patient. This may lead to the ability to perform asleep MRgFUS, enhancing patient comfort and increasing access to this life-changing therapy.

## Data Availability

The raw data supporting the conclusions of this article will be made available by the authors, without undue reservation.

## Supplementary material

Supplementary Figure S1 Relationship between NTC and CRST scores. Higher NTC was weakly correlated with CRST score reduction for CRST part A (treated hand only), part B (treated hand only, no pouring), and part C (functional assessment only). CRST: Clinical Rating Scale for Tremor; NTC: normative tractography coefficient for VIM-precentral hotspot.

Supplementary Figure S2 CRST B scores vs. length of time to last follow-up. (a) Patients with longer follow-up do not have less tremor improvement at last follow-up as measured by reduction in CRST part B (treated hand only, no pouring) scores. (b) This is also true when considering only subjects with smaller lesions (lesion volume < 127.4 mm^3^, the cutoff below which patients are more likely to have “better” or “approximately the same” QoL at last follow-up). This suggests that most patients, including those with smaller lesions, did not have tremor recurrence during the study period, but future work looking at longer follow-up (e.g., > 5 years) is merited. CRST: Clinical Rating Scale for Tremor.
