# Supplementary figures and images for: “Quality over quantity:” smaller, targeted lesions optimize quality of life outcomes after MR-guided focused ultrasound thalamotomy for essential tremor
Source: Front Neurol. 2024 Nov 13;15:1450699. doi: 10.3389/fneur.2024.1450699 (PMC11603361; doi:10.3389/fneur.2024.1450699)

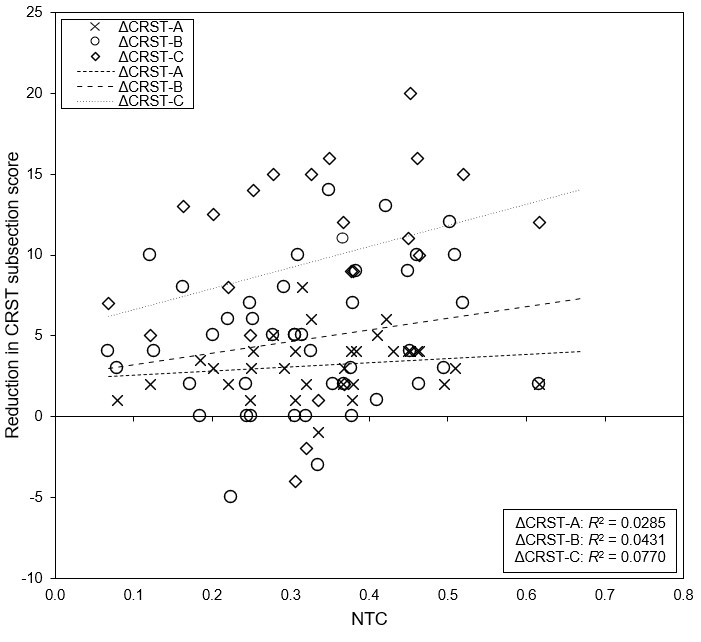

Supplement: Supplementary Figure S1 — Relationship between NTC and CRST scores. Higher NTC was weakly correlated with CRST score reduction for CRST part A (treated hand only), part B (treated hand only, no pouring), and part C (functional assessment only). CRST: Clinical Rating Scale for Tremor; NTC: normative tractography coefficient for VIM-precentral hotspot. [file Image_1.jpg]

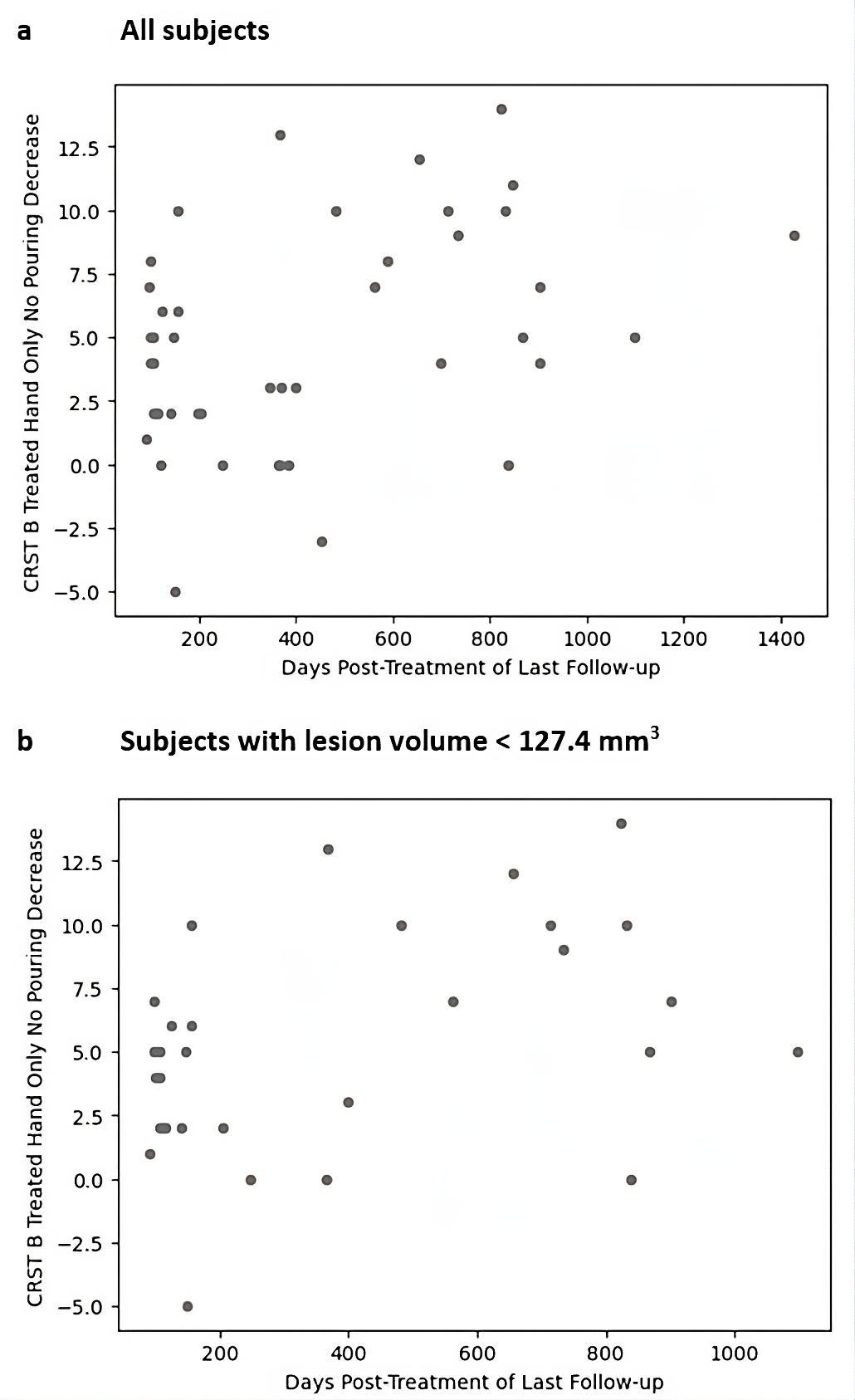

Supplement: Supplementary Figure S2 — CRST B scores vs. length of time to last follow-up. (a) Patients with longer follow-up do not have less tremor improvement at last follow-up as measured by reduction in CRST part B (treated hand only, no pouring) scores. (b) This is also true when considering only subjects with smaller lesions (lesion volume < 127.4 mm3, the cutoff below which patients are more likely to have “better” or “approximately the same” QoL at last follow-up). This suggests that most patients, including those with smaller lesions, did not have tremor recurrence during the study period, but future work looking at longer follow-up (e.g., > 5 years) is merited. CRST: Clinical Rating Scale for Tremor. [file Image_2.jpeg]
